# Hardware Implementation-Based Lightweight Privacy- Preserving Authentication Scheme for Internet of Drones Using Physically Unclonable Function

**DOI:** 10.3390/s26072224

**Published:** 2026-04-03

**Authors:** Razan Alsulieman, Eduardo Hernandez Escobar, Richard Swilley, Ahmed Sherif, Kasem Khalil, Mohamed Elsersy, Rabab Abdelfattah

**Affiliations:** 1 School of Computing Sciences and Computer Engineering, University of Southern Mississippi, Hattiesburg, MS 39406, USA; razan.alsulieman@usm.edu (R.A.); eduardo.a.hernandez@usm.edu (E.H.E.); richard.swilley@usm.edu (R.S.); rabab.abdelfattah@usm.edu (R.A.); 2 Electrical and Computer Engineering Department, University of Mississippi, Oxford, MS 38677, USA; kmkhalil@olemiss.edu; 3 Department of Electrical Engineering, Assiut University, Assiut 71515, Egypt; 4 Computer Information Systems Department, Higher Colleges of Technology, Al Ain 25026, United Arab Emirates; melsersy@hct.ac.ae

**Keywords:** Internet of Drones, physical unclonable function, authentication, hardware acceleration

## Abstract

The Internet of Drones (IoD) has emerged as a critical extension of the Internet of Things, enabling unmanned aerial vehicles to support diverse applications, including precision agriculture, logistics, disaster monitoring, and security surveillance. Despite its rapid growth, securing IoD communications remains a significant challenge due to the open wireless environment, high drone mobility, and strict computational and energy constraints. Existing authentication mechanisms either rely on computationally expensive cryptographic operations or remain validated only at the protocol or simulation level, leaving a critical gap in practical, hardware-validated solutions suitable for resource-constrained drone platforms. This gap motivates the need for a lightweight, privacy-preserving authentication scheme that is both theoretically sound and experimentally deployable on real hardware. To address this, we propose a Physically Unclonable Functions (PUF)-assisted lightweight authentication scheme for IoD environments that binds cryptographic keys to each drone’s intrinsic hardware characteristics via PUFs. The scheme employs dynamically generated pseudo-identities to conceal permanent drone identities and prevent tracking, while authentication and key agreement are achieved using efficient symmetric cryptographic primitives, including SHA-256 for key derivation and updates, AES-256 for secure communication, and lightweight XOR operations to minimize overhead. Forward secrecy is ensured through rolling key updates, and periodic renewal of PUF challenges enhances resistance to replay and modeling attacks. To validate practicality, both software-based and hardware-based implementations were developed and evaluated. The software evaluation demonstrates a low communication overhead of 708.5 bytes and an average computation time of 18.87 ms. The hardware implementation on a Nexys A7-100T FPGA operates at 100 MHz with only 12.49% LUT utilization and low dynamic power consumption of approximately 182.5 mW. These results confirm that the proposed framework achieves an effective balance between security, privacy, and efficiency. The significance of this work lies in providing a fully hardware-validated, PUF-based authentication framework specifically tailored to the real-world constraints of IoD environments, offering a practical foundation for securing next-generation drone networks.

## 1. Introduction

The Internet of Drones (IoD) has rapidly transitioned from military applications to civilian and commercial domains owing to its versatility across a wide range of use cases, including natural-disaster monitoring, disease-spread tracking, traffic management, and security surveillance [1]. As drone usage continues to diversify, advancements in emerging technologies, reductions in manufacturing costs, the global shift toward Industry 4.0, and the rapid development of the Internet of Things (IoT) ecosystem are significantly accelerating the growth of IoD networks [2]. Despite these advancements, the security and privacy of IoD systems remain major concerns, as they are increasingly targeted by sophisticated cyberattacks [3]. Establishing robust cybersecurity mechanisms has therefore become imperative as drones play a more critical role in safety- and mission-critical infrastructures, aligning with the broader evolution toward intelligent, secure, and highly interconnected systems.

Although significant progress has been made in IoD security, many existing authentication protocols fail to adequately balance strong security guarantees with resource efficiency. The open and dynamic nature of drone communication channels exposes IoD networks to numerous cyber threats, including eavesdropping, impersonation, data manipulation, and unauthorized access [4,5]. This risk is further amplified by the possibility of drones being hijacked or repurposed for malicious activities, underscoring the urgent need for reliable and resilient authentication mechanisms. In parallel, several studies have explored drone identification using physical-layer features, such as RF signal analysis, vibration-based signatures, and machine-learning-based recognition techniques [6,7,8]. More recent work has also investigated physical fingerprint-based authentication in IoT environments [9]; however, such approaches are not specifically tailored to the constraints of IoD systems.

However, because drones are constrained by limited computational power, memory capacity, and battery life, lightweight authentication schemes are essential for IoD environments where traditional cryptographic solutions may impose excessive overhead [10,11,12,13]. In contrast, lightweight protocols minimize computational and communication costs while ensuring essential security properties, such as mutual authentication, data integrity, and resistance to common attacks, making them suitable for large-scale, energy-constrained IoD networks [14].

Modern cryptographic protocols rely heavily on secure hash functions to guarantee data integrity, authentication, and secure key derivation. Among these functions, SHA-256 from the SHA-2 family is widely adopted in security systems due to its strong resistance to collision and preimage attacks while maintaining efficient computational performance [15]. SHA-256 is commonly used in applications such as digital signatures, secure communication protocols, blockchain systems, and authentication mechanisms, making it a fundamental component of modern cybersecurity infrastructures. In IoT and IoD environments, SHA-256 is commonly used for lightweight key derivation and session key updates [16]. In addition, researchers have increasingly explored hardware implementations of cryptographic primitives on Field-Programmable Gate Arrays (FPGAs) to improve performance and energy efficiency [17]. FPGA platforms enable parallel processing and customizable hardware architectures, significantly accelerating cryptographic algorithms compared with software implementations [17,18]. Several studies have demonstrated that FPGA implementations of SHA-256 can achieve high throughput while maintaining low resource utilization and power consumption, making them well-suited for embedded and resource-constrained security applications [19,20]. Motivated by these advantages, the proposed scheme integrates SHA-256 for lightweight key derivation and rolling session key updates, and employs FPGA-based hardware acceleration to implement the PUF-assisted authentication framework on the Nexys A7-100T platform. This hardware–software co-design enables efficient, practical, and secure authentication tailored specifically to the computational and energy constraints of resource-constrained IoD environments.

Prior research on hardware-assisted authentication for the IoD remains limited and fragmented. Researchers in [21] implemented a privacy-aware authentication scheme using FPGA-based hardware acceleration, thereby demonstrating the feasibility of offloading security operations to hardware; however, their design relies on machine-learning components and encryption-intensive processing, which increases system complexity and resource overhead, potentially limiting scalability for lightweight drone platforms. Similarly, the study of [22] presented Physically Unclonable Function (PUF)-based privacy-preserving authentication protocols with hardware considerations for IoT devices; nevertheless, this work targets general IoT environments and does not explicitly address the mobility, latency, and energy constraints unique to IoD systems. In contrast, the work in [23] remains primarily at the protocol and simulation levels, lacking full hardware realization and detailed resource and power analyses. Moreover, researchers in [24], through a comprehensive survey, emphasized that while PUFs offer strong potential for trusted IoD authentication, practical hardware implementations that simultaneously evaluate security, efficiency, and real-world deployability remain scarce. Consequently, these limitations motivate the need for a fully implemented, experimentally validated hardware-based authentication scheme tailored to IoD environments.

In this paper, we propose a PUF-assisted lightweight authentication scheme for IoD that integrates key generation, authentication, and key updates into a unified design. Drone identities are protected using nonce-based pseudo-IDs, while a hardware-embedded PUF generates unique challenge–response pairs to bind cryptographic keys to each drone’s hardware. Seed keys are derived using SHA-256, and subsequent authentication relies on lightweight AES encryption and hash-based key evolution to ensure secure communication, anonymity, and forward secrecy with low computational and communication overhead. To demonstrate practical deployability, the scheme is evaluated through both software and hardware implementations. A Python-based implementation assesses protocol efficiency, while a full hardware realization is performed on the Nexys A7-100T FPGA using Xilinx Vivado 2023.2. The design integrates a Hybrid XOR–RO PUF, AES, SHA-256, and lightweight control logic operating at 100 MHz, enabling accurate evaluation of area, power, and latency. The hardware-based PUF provides unclonable and reliable identities, confirming the suitability of the proposed scheme for real-world, resource-constrained IoD environments. Hardware implementation on a Nexys A7-100T FPGA further demonstrates the practicality of the proposed approach, achieving only 12.49% LUT utilization, 7.37% register usage, and stable operation at 100 MHz with low power consumption.

To validate the practicality of the proposed scheme, both software- and hardware-based implementations are evaluated. The software implementation demonstrates low computational and communication overhead, requiring only seven message exchanges and incurring a total communication cost of 708.5 bytes, while achieving an average computation time of 18.87 ms. These results indicate that the proposed authentication protocol is efficient and suitable for deployment in large-scale IoD networks. In addition, a hardware implementation on an FPGA platform confirms the feasibility of integrating the proposed cryptographic primitives into resource-constrained systems, achieving low execution latency and moderate power consumption. The comparative analysis highlights that while the software implementation is effective for protocol validation and scalability analysis, the hardware implementation provides clear advantages in terms of execution efficiency and energy suitability, making it more appropriate for real-time and mission-critical IoD applications.

In our prior work [25], we investigated both hash-based and chaotic-map-based authentication schemes for IoD environments. However, these approaches did not incorporate hardware-rooted security primitives such as PUFs, nor were they fully validated on FPGA implementations.

The main contributions of this work are summarized as follows:

- **A lightweight and privacy-preserving authentication framework for IoD:** A privacy-preserving authentication scheme that combines PUF-based hardware identities, pseudo-identifiers, and symmetric cryptography to secure resource-constrained drone networks.
- **A Hybrid XOR–Ring Oscillator (XOR–RO) PUF architecture:** A novel PUF design that improves uniqueness, reliability, and resistance to modeling attacks while remaining suitable for FPGA-based embedded systems.
- **Hardware-software implementation:** The proposed framework is implemented and evaluated using both software analysis and FPGA realization on a Nexys A7-100T platform that integrates PUF, SHA-256, and AES-256.
- **Demonstration of efficient and practical security for resource-constrained IoD systems:** Experimental results demonstrate low communication overhead, efficient computation, moderate FPGA resource usage, and low power consumption.

The remainder of this paper is organized as follows. Section 2 reviews existing authentication protocols and summarizes related work in the context of the IoD. Section 3 describes the system model, including the network model, adversary model, and the design goals of the proposed scheme. Section 4 introduces the necessary background concepts, including PUFs, cryptographic hash functions, and the AES encryption scheme. The proposed PUF-based authentication scheme, encompassing the registration, PUF-based key generation, authentication, and key update phases, is presented in Section 5. Section 6 provides a comprehensive evaluation of the proposed scheme, including security and privacy analysis as well as performance assessment through both software and hardware implementations, and an in-depth evaluation of the proposed Hybrid XOR–RO PUF. Finally, Section 8 concludes the paper by summarizing the key findings and contributions.

## 2. Related Work

Authentication in IoD has been extensively investigated, with researchers proposing solutions based on lightweight cryptography, privacy-preserving mechanisms, decentralized trust, and hardware-assisted security [21,26,27]. Early efforts primarily focused on software-based lightweight authentication schemes to accommodate the limited resources of drones. For example, Ref. [28] introduced SLPAKA, a certificate-free authenticated key agreement scheme based on elliptic curve cryptography, demonstrating resistance to several known attacks under the Canetti–Krawczyk adversary model. Similarly, in [29], researchers proposed an HMAC-SHA1-based authentication protocol for flying ad hoc IoD networks, while SELTHA [30] employed hash-based and Time-based One-Time Password (TOTP) mechanisms for mutual authentication. Although these approaches reduce computational overhead, they rely entirely on software-based cryptography and often incur higher communication costs or provide limited privacy protection, which restricts their applicability in highly dynamic IoD environments.

To further enhance trust and security, blockchain-assisted authentication schemes have also been explored. Researchers in [31] proposed a blockchain-based lightweight authentication service for industrial UAVs, and ref. [32] later integrated blockchain with PUFs for UAV-enabled intelligent transportation systems. While these schemes improve decentralization and auditability, they introduce additional latency and communication overhead, which can negatively impact scalability and real-time performance in resource-constrained drone networks. Previous work has explored lightweight authentication schemes for IoD using hash-based mechanisms such as HMAC and SHA-256 [25]. Previous work also explored chaotic-map-based approaches leveraging Chebyshev polynomials [33]. While these methods achieve low computational overhead, they either lack hardware validation or rely on primitives with limited standardization and cryptographic assurance.

While this paper and [25] use PUFs and hardware acceleration for authentication, they address different problem domains and make different contributions from a cryptographic and hardware perspective. From a cryptographic perspective, this paper employs a challenge–response authentication scheme to verify user legitimacy. This is done by knowing some secret information that will be used in a response to a challenge. However, in [25], we used a searchable encryption technique to search over encrypted data (identities) with multi-entity matching using a centralized server to provide the authentication. From a hardware perspective, this paper presents a substantially different PUF architecture than our work in [25]. In this paper, we propose a novel hybrid XOR-RO PUF design that integrates Arbiter-based and Ring Oscillator structures to enhance entropy and security performance. In contrast, our earlier publication employed a heterogeneous PUF framework that combined delay-based PUF, RO-PUF, and SRAM-PUF mechanisms. Also, this paper introduces a new architectural configuration and design methodology, focusing specifically on hybrid XOR integration between Arbiter and RO components, which was not explored in [25]. Our scheme in this paper introduces hash-chain-based key evolution, periodic PUF challenge renewal, dynamic pseudo-identities, and a Hybrid XOR–RO PUF with full FPGA implementation. Therefore, our contribution differs significantly in system model, protocol design, and security mechanisms, focusing on real-time, lightweight authentication for resource-constrained drone environments.

In contrast, PUFs have attracted increasing attention as a lightweight, privacy-preserving authentication primitive for IoD. The work in [10] proposed PMAP, a PUF-based mutual authentication protocol that incorporates chaotic systems to enhance randomness and conditional privacy, followed by liteGAP [34], which supports lightweight group authentication using hash functions, XOR operations, and PUFs. Researchers in [35,36] similarly combined chaotic maps with PUFs to improve resistance against impersonation and cloning attacks, though at the cost of increased system complexity. Furthermore, the study in [37] introduced SLAP-IoD, a PUF-based authentication protocol that employs a fuzzy extractor, whereas researchers in [13,38,39] proposed lightweight PUF-based authentication and key agreement schemes using hash and XOR operations. Collectively, these studies demonstrate the effectiveness of PUFs in enhancing IoD security and privacy.

Nevertheless, despite their promising security properties, most existing PUF-based IoD authentication schemes have been validated only at the protocol or simulation level and assume ideal PUF behavior. In addition, few studies provide a comprehensive evaluation of hardware-related factors, including resource utilization, power consumption, reliability under environmental variations, and real-time performance. Other optimization-oriented approaches, such as ODIAP [40], aim to reduce UAV-side computation by offloading complex operations to infrastructure nodes; however, these designs do not provide hardware-rooted device identities and depend heavily on network support.

In addition to cryptographic authentication approaches, several works have investigated drone identification using physical-layer characteristics. These include RF-based identification, vibration-based fingerprinting, and deep learning-based signal classification techniques [6,7,8], which leverage inherent hardware features to distinguish drones. However, these approaches do not provide mutual authentication, secure session key establishment, or resistance to network-level attacks. More recently, hybrid physical fingerprint-based authentication mechanisms have been proposed for IoT systems [9], but these schemes are not specifically designed for highly dynamic IoD environments and lack lightweight hardware-integrated authentication frameworks.

While prior research has significantly advanced lightweight and PUF-based authentication for IoD, clear gaps remain in terms of practical deployability and hardware validation. In particular, the lack of fully implemented and experimentally evaluated hardware-based PUF authentication schemes motivates the present work, which focuses on a lightweight, privacy-preserving authentication framework with a complete FPGA-based PUF implementation, tailored to the real-world constraints of IoD environments.

## 3. System Model and Design Goals

### 3.1. Network Model

**Figure 1:**
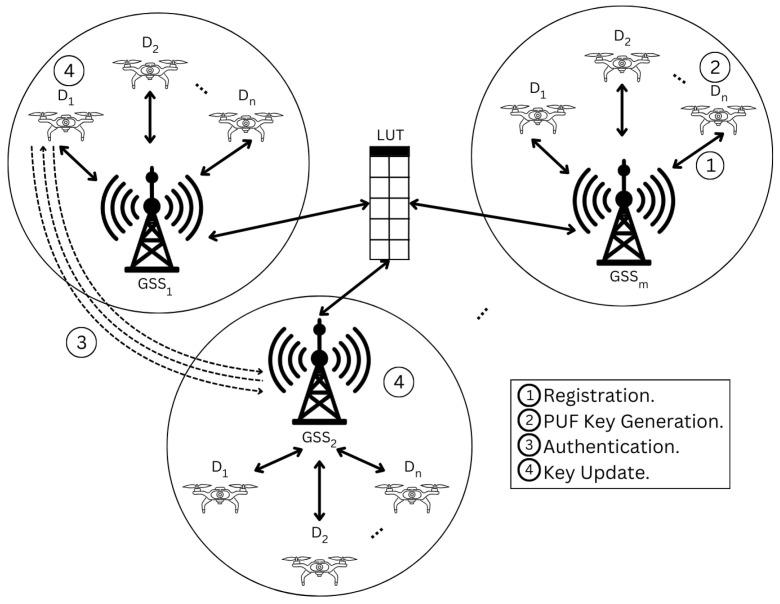
Network Model.

The network model, as illustrated in Figure 1, comprises drones and Ground Station Servers (GSSs) that work in coordination to ensure secure and efficient operations. Each drone operates as an independent device, equipped with a unique ID for identity verification. These drones, equipped with built-in security mechanisms, perform tasks within designated field zones and continuously authenticate to preserve network integrity. Secure communication protocols prevent unauthorized access, ensuring that data transmission between drones and the network remains protected. The GSSs act as a central controller and facilitator of the network. GSSs manage drone registration, authenticate drones using their PUFs, and retrieve critical operational data from them. Within this hierarchical framework, the GSSs ensure that drones operate efficiently and securely within their assigned zones, thereby maintaining uninterrupted network functionality.

The communication between drones and GSSs is assumed to be established over standard wireless technologies commonly used in IoD environments, such as Wi-Fi, cellular networks (4G/5G), or Flying Ad Hoc Networks (FANETs) [41,42]. These technologies provide reliable bidirectional communication and sufficient bandwidth for authentication and data exchange. The proposed authentication protocol operates at the application/security layer and is independent of the underlying communication technology, ensuring compatibility with existing drone communication hardware and networking stacks. The proposed authentication mechanism operates above the underlying wireless communication stack and therefore does not alter the normal operation of drone communications at the PHY, MAC, routing, or transport layers. Consequently, deployment-dependent effects such as channel-access delay, retransmissions, routing overhead, and propagation latency remain unchanged by the proposed scheme and are common to any secure communication system built over the same wireless technology. In this work, the communication analysis isolates only the incremental protocol overhead introduced by the proposed authentication operations. Furthermore, the protocol’s lightweight design and low communication overhead make it suitable for deployment on resource-constrained UAV platforms.

### 3.2. Threat Model

In the proposed network, communication over insecure wireless channels introduces significant risks, as entities cannot always be presumed trustworthy. The adversary’s objectives include gaining unauthorized access to the network, disrupting operations, stealing sensitive information, or injecting malicious messages into the communications exchanged between drones and the GSSs. In extreme scenarios, such attacks could target critical infrastructure or government assets, with drones potentially weaponized against strategic sites, posing a significant threat to national security. Drones operating in hostile or high-risk environments are more vulnerable, particularly when carrying sensitive data. The adversary is assumed to know the protocol structure and to have access to public information, such as drone IDs and timestamps, but is constrained by their inability to break cryptographic primitives such as AES-256 or SHA-256, clone PUFs, or compromise long-term shared keys. These assumptions ensure that the proposed scheme is evaluated against realistic, well-defined threats, thereby highlighting its robustness in securing IoD networks.

In addition to network-level attacks, we also consider the possibility that an adversary may physically capture a drone and attempt to extract stored secrets, including the pre-shared key $K_{s}$, using hardware probing or side-channel techniques. In the proposed scheme, the pre-shared key $K_{s}$ is used only during the initial registration phase to establish a secure channel between the drone and the GSS. Subsequent authentication relies on session keys derived from PUF-generated responses, which are tied to the unique physical characteristics of the drone hardware and cannot be reproduced on another device. However, we assumed that the secret key, $K_{s}$, that will be used in the symmetric key encryption in the registration step, will be stored in a secure memory, such as SRAM. In addition, even if the attacker has physical access to the drone, they cannot obtain the PUF response, as the PUF design is unknown to them.

### 3.3. Design Goals

The proposed scheme aims to provide the following security features.

#### 3.3.1. Robust Authentication

The proposed scheme should ensure reliable authentication. Only legitimate drones can contribute to the system functionality, effectively preventing impersonation attacks. All authentication messages should be protected against message modifications or replay attacks. In addition, unauthorized entities are prevented from accessing the network resources.

#### 3.3.2. Scalability

The proposed scheme should be designed to scale efficiently with the growth of IoD networks. Lightweight operations should be used without increasing computation overhead to support large-scale deployments.

#### 3.3.3. Confidentiality

Confidentiality of transmitted data, such as sensitive messages, pseudo-identities, nonces, and PUF responses, should be maintained. This prevents eavesdropping or traffic analysis attacks.

#### 3.3.4. Privacy Preservation

The proposed scheme should ensure drone privacy by concealing its real identity. By avoiding the transmission of real drone IDs and enforcing session unlinkability, the scheme prevents identity disclosure and long-term tracking.

## 4. Preliminaries

### 4.1. Physical Unclonable Function (PUF)

Similar to an individual’s biometric information, a PUF serves as a unique hardware identity for each drone. In the IoD, PUFs arise from inherent nanoscale manufacturing variations in integrated circuits (ICs), making it infeasible to clone or reproduce the same PUF behavior on another device. An ideal PUF provides essential security properties such as uniqueness, reliability, and unpredictability, while offering strong resistance against cloning and physical tampering attacks. Mathematically, a PUF can be expressed as R=P(C), where *C* represents the input challenge, P(·) denotes the PUF function, and *R* is the generated response. Since the output depends on the intrinsic physical characteristics of the IC, any physical modification to the drone hardware alters the PUF response, thereby revealing tampering attempts [37].

### 4.2. Cryptographic Hash Function (SHA-256)

SHA-256 plays a critical role in authenticating key-exchange protocols by balancing security and efficiency. It converts input data of arbitrary length into a fixed-length 256-bit hash and performs 64 rounds of processing to ensure strong cryptographic security, thereby making it resistant to brute-force and collision attacks, which is essential for securing drone communications [43,44]. SHA-256 enhances security over its predecessors, such as SHA-1 and MD5, by addressing known vulnerabilities, including collision attacks, and is widely used for authentication and key derivation in IoD networks [45].

### 4.3. Advanced Encryption Standard (AES-256)

The AES is a symmetric block cipher widely used to secure data in resource-constrained systems, including IoT and IoD environments [46,47]. AES operates on fixed-size 128-bit data blocks and supports key lengths of 128, 192, and 256 bits; AES-256 provides the highest security level with 14 rounds. AES-256 offers a strong balance between cryptographic security and computational efficiency, making it suitable for devices with limited processing power and energy resources. Its resistance to known cryptanalytic attacks and support for efficient hardware and software implementations have led to its widespread use in security-critical and embedded applications [48]. As a result, AES-256 is commonly employed to ensure the confidentiality and integrity of data transmitted over insecure wireless channels in IoD networks.

## 5. Proposed Authentication Scheme

This scheme proposes an authentication scheme combining key generation and authentication between drones and the ground station. It leverages a PUF for creating challenge–response pairs, ensuring secure communication. The proposed scheme employs AES-256 to encrypt communications between drones and the GSS while SHA-256 is used to derive seed and session keys from pseudo-identities and PUF-based values, ensuring that cryptographic keys are bound to both hardware characteristics and fresh protocol inputs. Furthermore, hash-based rolling key updates provide forward secrecy, ensuring that compromise of a current session key does not expose past communications. Using nonces with SHA-256 ensures message freshness and prevents replay attacks. The proposed scheme comprises several subsections: PUF design, registration, key generation, the authentication phase, and key update.

### 5.1. PUF Design

**Figure 2:**
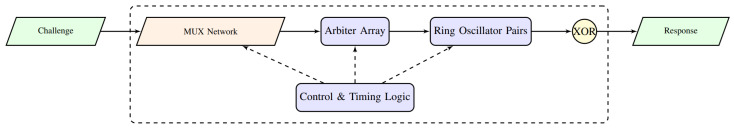
The hybrid XOR-RO PUF architecture.

Building on these principles, the proposed Hybrid XOR-Ring Oscillator (XOR-RO) PUF architecture combines delay-based and frequency-based entropy sources to achieve high uniqueness, stability, and resilience against modeling attacks. Traditional Arbiter PUFs provide strong challenge–response randomness but may suffer from reduced reliability under environmental variations, whereas Ring Oscillator (RO) PUFs offer robust temporal entropy derived from frequency differences but can become predictable when implemented with limited resources. To address these limitations, the proposed design integrates multiple arbiter chains with frequency-controlled RO modules to capture both delay and frequency variations at the hardware level, as illustrated in Figure 2.

The architecture consists of four main components: a challenge MUX network, an arbiter array, a ring oscillator pair bank, and an XOR response combiner. The challenge vector is first applied to the MUX network, which dynamically selects signal paths through the arbiter array. Each arbiter chain generates a binary response based on intrinsic delay differences caused by manufacturing process variations. These outputs then control the corresponding RO pairs, in which frequency differences are measured using counters and converted into digital bits. Finally, the outputs of all arbiter-RO pairs are XOR-combined to generate the final PUF response. A dedicated control and timing logic unit coordinates signal sequencing, synchronization, and readout, enabling efficient operation on FPGA hardware. This hybrid design significantly enhances response unpredictability and reliability. The XOR combination of arbiter and RO outputs improves resistance to machine learning-based modeling attacks, while the frequency-based RO layer mitigates the effects of temperature and voltage fluctuations. Moreover, the architecture is fully synthesizable using standard FPGA primitives such as LUTs, registers, and counters, ensuring low resource utilization and scalability. As implemented on the Nexys A7-100T FPGA, the proposed Hybrid XOR-RO PUF exhibits strong entropy characteristics, low power consumption, and seamless integration with the AES and SHA-256 cores within the proposed authentication framework.

### 5.2. Registration

During the registration phase of the proposed authentication scheme, several key assumptions and prerequisites are established to ensure the protocol’s effective implementation. We assume that the long-term shared key $K_{s}$ is securely created and shared between the drone and the GSS during drone registration, where it is used only during the registration phase to establish a secure initial communication channel with preserving the privacy of the drone by hiding its real identity in the first step of the registration step. After the registration process is completed and the PUF-derived seed key $K_{0}$ is generated, subsequent authentication and session key generation rely exclusively on the PUF-derived keys rather than the master key. As a result, the exposure window of $K_{s}$ is minimized. If a drone is reinitialized or re-enrolled into the network, a new master key can be securely provisioned during the re-registration process. This lifecycle management ensures that the master key is used only for bootstrapping trust and does not participate in long-term authentication operations. Authentication relies on PUF-derived keys that bind cryptographic operations to each drone’s physical hardware. Unlike conventional approaches that depend solely on stored secret keys, the use of a PUF provides a hardware-rooted identity that cannot be cloned or replicated, thereby strengthening resistance to device cloning and impersonation attacks. Also, implement any symmetric encryption with $K_{s}$ in hardware, which ensures the low computation overhead and can also be made offline and before the actual authentication step.

**Figure 3:**
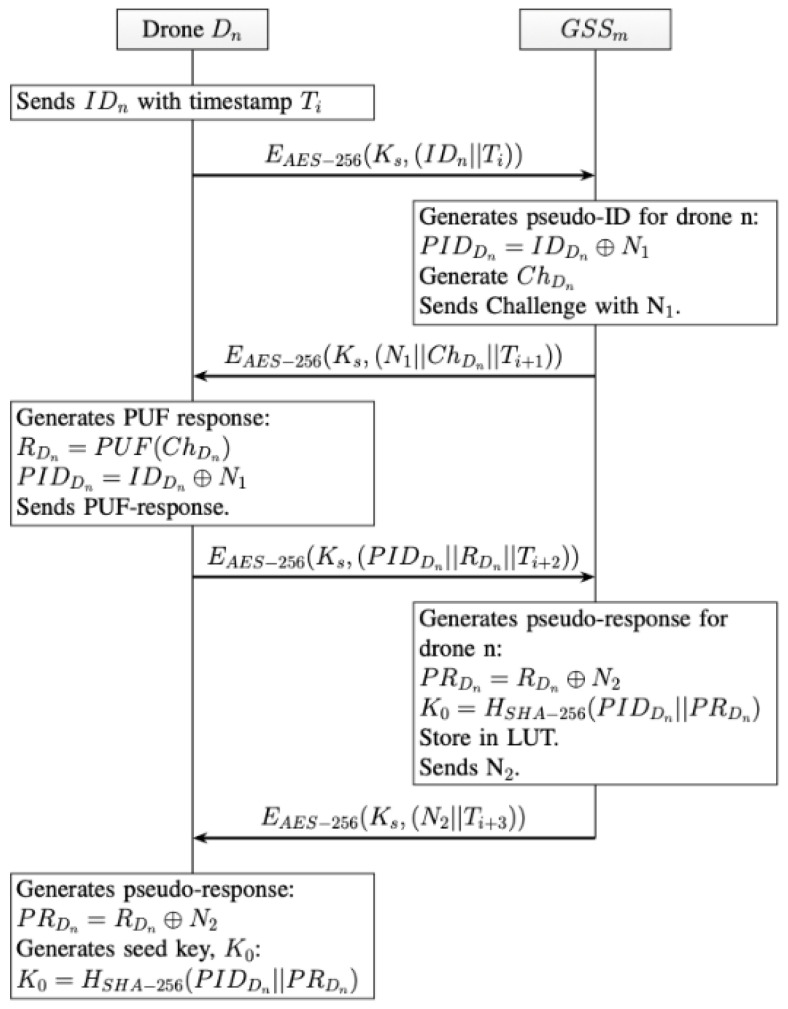
Registration Phase Communication Scheme.

$K_{s}$ serves as the foundation for secure initial communication and is used to encrypt sensitive information such as the drone’s unique identifier $ID_{n}$ and timestamps $T_{i}$. As summarized in Figure 3, each drone is equipped with a unique, consistent, and noise-free PUF value that serves as its secure identity and authentication foundation. The GSS maintains a predefined list of authorized drones within the IoD network, ensuring only trusted devices can communicate, thereby mitigating the risk of unauthorized access.

Initially, the drone ($D_{n}$) sends its ID with timestamp $T_{i}$ encrypted with long-term shared key $K_{s}$:

$$E_{AES-256}(K_{s},(ID_{n}\left|\right|T_{i}))$$

The GSS generates the pseudo-ID for drone ($D_{n}$) using a nonce, $N_{1}$:

$$PID_{D_{n}}=ID_{D_{n}}⊕N_{1}$$

The GSS then generates a PUF challenge ${\mathrm{Ch}}_{D_{n}}$, using our proposed PUF in Section 5.1, and sends a challenge to the drone along with nonce, $N_{1}$, and timestamp, $T_{i+1}$:

$$E_{AES-256}(K_{s},(N_{1}||Ch_{D_{n}}\left|\right|T_{i+1}))$$

Drone ($D_{n}$) generates the PUF response, $R_{D_{n}}$:

$$R_{D_{n}}=PUF\left(Ch_{D_{n}}\right)$$

Drone ($D_{n}$) sends pseudo-ID, PUF response, and timestamp, $T_{i+2}$, back to the GSS:

$$E_{AES-256}(K_{s},(PID_{D_{n}}\left|\right|R_{D_{n}}\left|\right|T_{i+2}))$$

The GSS generates a pseudo-response for drone ($D_{n}$) using a nonce, $N_{2}$:

$$PR_{D_{n}}=R_{D_{n}}⊕N_{2}$$

Then, the GSS generates a seed key for Drone ($D_{n}$):

$$K_{0}=H_{SHA-256}(PID_{D_{n}}||PR_{D_{n}})$$

All GSSs store the pseudo-ID, $PID_{D_{n}}$, and PUF pseudo-response, $PR_{D_{n}}$, for each drone, in a Look-Up-Table (LUT). The GSS sends nonce, $N_{2}$, and timestamp, $T_{i+3}$, to drone ($D_{n}$):

$$E_{AES-256}(K_{s},(N_{2}\left|\right|T_{i+3}))$$

Finally, drone ($D_{n}$) calculates its pseudo-response, $PR_{D_{n}}$, with its own PUF response, and $N_{2}$, using XOR operation. Drone ($D_{n}$) calculates its seed key by using a hash function of its pseudo-ID, $PID_{D_{n}}$, concatenated with its pseudo-response, $PR_{D_{n}}$.

It is worth noting that the registration phase is a one-time procedure, performed only during initial drone deployment or scheduled maintenance, when stable, sustained connectivity can be assumed. As a result, the four-message exchange incurred during registration does not affect real-time communication performance in highly dynamic flight environments. Once registration is complete, all subsequent interactions rely exclusively on the lightweight authentication phase, which requires only three message exchanges and significantly lower communication overhead, making it well-suited to brief, unstable wireless connection windows typical of IoD operations.

### 5.3. PUF-Based Key Generation

The PUF-Based Key Generation process is a core component of the proposed authentication scheme. This ensures that only the legitimate drone can generate the correct response. In the proposed design, the PUF is used to derive a device-specific, random secret key rather than a random identity. Drone identities are protected separately using dynamically generated pseudo-identifiers, while the PUF provides entropy for secure key generation. The key generation process begins with a PUF challenge–response mechanism. Each drone is equipped with an embedded PUF that produces a unique response based on a given challenge. When the GSS sends a challenge $Ch_{D_{n}}$ to the drone $D_{n}$, the drone evaluates its PUF to generate a response $R_{D_{n}}$. This response is unpredictable and cannot be reproduced by any other device, ensuring that only the legitimate drone can generate the correct PUF output.

To enhance security and prevent direct exposure of raw PUF responses, the GSS generates a pseudo-response $PR_{D_{n}}$ by XORing the PUF response with a fresh nonce $N_{2}$:

$$PR_{D_{n}}=R_{D_{n}}⊕N_{2}$$

This masking operation ensures that the PUF response is never transmitted or stored in its original form. The masked PUF output is then combined with the drone’s $PID_{D_{n}}$, which is itself generated using nonce-based XOR operations to conceal the real drone identity. A seed key $K_{0}$ is derived by applying the SHA-256 hash function to the concatenation of $PID_{D_{n}}$ and $PR_{D_{n}}$:

$$K_{0}=H_{\mathrm{SHA}-256}\left(PID_{D_{n}}\;∥\;PR_{D_{n}}\right)$$

The resulting seed key is a PUF-derived random secret that forms the basis for subsequent session key generation and rolling key updates used during authentication.

The PUF-based key generation process is designed to be both secure and lightweight, making it suitable for resource-constrained drone environments. By using the PUF to generate cryptographic keys rather than identities, the scheme achieves strong resistance to impersonation and cloning attacks while preserving privacy through pseudo-identifiers. Moreover, the reliance on simple XOR operations and hash functions minimizes computational and energy overhead, enabling efficient deployment on drones with limited processing capabilities and battery life.

### 5.4. Authentication Phase

**Figure 4:**
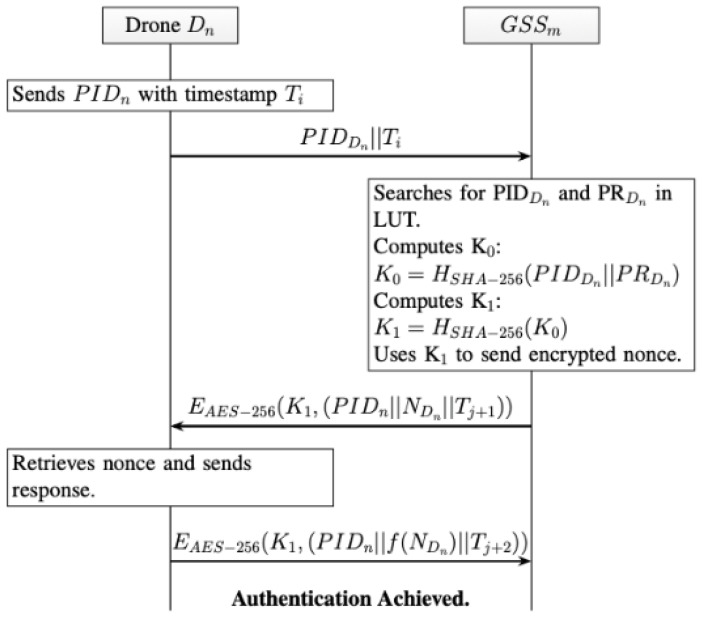
Authentication Phase Communication Scheme.

As illustrated in the communication flow summarized in Figure 4, to establish authentication, the drone ($D_{n}$) initiates communication by sending its $PID_{D_{n}}$ and timestamp, $T_{j}$, to any GSS:

$$PID_{D_{n}}\left|\right|T_{j}$$

The GSS searches for the $PID_{n}$ and $PR_{D_{n}}$ in the LUT. The GSS computes the seed key, $K_{0}$, of drone, $D_{n}$, using the hash function of the $PID_{n}$ and $PR_{D_{n}}$. Both drone, $D_{n}$ and GSS compute $K_{1}$:

$$K_{1}=H_{SHA-256}\left(K_{0}\right)$$

The GSS then uses $K_{1}$ to send an encrypted nonce, $N_{D_{n}}$, with $PID_{n}$, and timestamp, $T_{j+1}$:

$$E_{AES-256}(K_{1},(PID_{n}\left|\right|N_{D_{n}}\left|\right|T_{j+1}))$$

Drone ($D_{n}$) would then retrieve the nonce and send a response with $PID_{D_{n}}$, a function of the nonce, $f(N_{D_{n}})$, and timestamp, $T_{j+2}$:

$$E_{AES-256}(k_{1},(PID_{n}||f\left(N_{D_{n}}\right)\left|\right|T_{j+2}))$$

This will achieve the authentication between the Drone ($D_{n}$) and GSS. To ensure robustness in unreliable wireless environments, which are especially common in highly dynamic IoD deployments where connection windows may be extremely brief, the proposed scheme includes a simple recovery mechanism to handle potential key desynchronization caused by packet loss or dropped connections. The GSS temporarily stores both the current and the previous session key for each drone. If an authentication attempt fails due to message loss or an interrupted connection, the server attempts verification using the previous key and timestamps, allowing the drone and the GSS to resynchronize without requiring re-registration. This mechanism ensures that transient connectivity disruptions during flight do not compromise authentication integrity or force the drone to restart the registration process. This design allows authentication to resume across intermittent connections without requiring a continuous communication session.

### 5.5. Key Update

To maintain long-term security, session keys are periodically updated using the hash of the previous key:

$$k_{x}=H_{SHA-256}\left(k_{n-1}\right)$$

The session key update mechanism follows a one-way hash-chain construction. Because SHA-256 is computationally one-way, an adversary who compromises a current session key cannot reverse it to recover any previously established session key, thereby protecting past communications. Furthermore, the seed key $K_{0}$, from which the entire key chain is derived, is computed from hardware-bound PUF responses and pseudo-identifiers. Since the PUF response is generated on demand from the drone’s physical hardware and is never stored in retrievable memory, an adversary cannot reconstruct the key chain origin without access to the legitimate physical PUF instance. In addition, periodic PUF challenges introduce fresh hardware-derived entropy that reseeds the key evolution process. Consequently, even if an adversary obtains the current session key $K_{n}$, which is rare and can be done only with the complete physical access of the drone, the attacker cannot predict future session keys beyond the current interval because the next key chain segment is derived from a new PUF response generated from the drone’s physical hardware. This mechanism limits the exposure window of any compromised session key to a single session interval. These mechanisms ensure that past session keys remain protected by the one-way hash construction, while future session keys are re-secured through periodic PUF challenge renewal.

## 6. Implementation and Performance Evaluation

To validate the practicality and efficiency of the proposed authentication scheme, both software-based and hardware-based implementations are developed and evaluated. While the software implementation demonstrates the protocol’s feasibility and light weight, the hardware implementation highlights its suitability for deployment in real-world, resource-constrained IoD environments. This section presents a comprehensive evaluation of the scheme with respect to security and privacy, software performance, and hardware efficiency.

### 6.1. Security and Privacy Analysis

This subsection evaluates the security and privacy properties of the proposed authentication scheme, demonstrating its robustness against common cyber threats while ensuring strong privacy protection for drones operating in IoD networks.

#### 6.1.1. Robust Authentication

The proposed authentication scheme ensures that only authorized drones can participate in the network by adopting a one-way authentication model in which the drone authenticates itself to the GSS. Authentication is achieved using a PUF-based challenge–response mechanism that binds the process to the drone’s unique and tamper-proof hardware characteristics, preventing unauthorized or cloned drones from accessing the network.

The authentication exchange is protected by nonces and session keys, which are encrypted with AES-256, ensuring the confidentiality of sensitive information such as nonces and PIDs. Furthermore, session keys derived using SHA-256 provide protection against impersonation and replay attacks, while the use of PIDs preserves drone privacy without compromising authentication reliability.

#### 6.1.2. Dynamic Key Generation

Dynamic session key generation is a critical feature of the scheme, enhancing forward security. During the registration phase, an initial session key is generated using PUF responses and cryptographic hashing. In subsequent communications, session keys are updated dynamically using *SHA-256*, ensuring that each session is unique and protected. Even if a session key is compromised, prior communications remain secure because of the forward security provided by this key update mechanism.

#### 6.1.3. Privacy Preservation

The scheme effectively protects drone privacy by using hashed PIDs rather than transmitting actual drone IDs. These PIDs are generated using *SHA-256* hashing and are unique for each session, making it impossible for adversaries to track drones across multiple sessions. Furthermore, encryption with *AES-256* ensures that sensitive information, including PIDs, nonces, and responses, remains inaccessible to unauthorized entities, thereby preserving data confidentiality and privacy.

#### 6.1.4. Resistance to Cyber Attacks

The proposed scheme is resilient against a wide range of cyber threats:

- Impersonation Attacks: The PUF-based challenge–response mechanism ensures that only genuine drones with unique hardware identities can authenticate successfully.
- Replay Attacks: The inclusion of nonces and timestamps in message exchanges prevents attackers from reusing old messages.
- Eavesdropping Attacks (Confidentiality): *AES-256* encryption and secure key exchange mechanisms protect against message interception and tampering.
- Key Compromise Attacks: Forward security ensures that past session communications remain secure even if a current session key is compromised.

#### 6.1.5. Efficient Resource Utilization

The protocol is designed for efficient operation using lightweight cryptographic primitives such as (*SHA-256*) hashing and (*AES-256*) encryption, which minimize computational overhead. The reliance on PUFs eliminates the need for complex key management, further reducing resource consumption. Communication overhead is also minimized by transmitting only essential encrypted information. The hardware realization further improves efficiency by performing security operations in dedicated modules that integrate the Hybrid XOR–RO PUF, AES, and SHA-256 at 100 MHz, thereby avoiding processor-intensive computation and relying primarily on simple logic operations. The PUF derives device-specific secrets without requiring secure key storage, thereby reducing memory overhead. As shown in Table 1, the FPGA results show moderate resource usage (12.49% LUTs, 7.37% registers, 8.88% block RAM) and low dynamic power (182.5 mW), supporting energy-constrained drone platforms.

#### 6.1.6. Scalability

The scheme is scalable to support a growing number of drones in the network. By using lightweight operations and pseudo-identifiers, the protocol avoids the need for large certificate databases or complex infrastructure. The use of LUT at the GSS for storing PIDs and session keys ensures efficient storage and retrieval, enabling seamless scalability as the number of drones increases.

### 6.2. Software Implementation

This section provides an in-depth analysis of the computational and communication overhead of the software implementation of the proposed authentication scheme, including the experiment setup and results, to demonstrate its efficiency and practicality in a mid-range computing environment.

#### 6.2.1. Experiment Setup

To evaluate the computation and communication costs of the proposed scheme, we implemented the scheme in Python 3.12.2 on a system with the following specifications: an HP ENVY x360 Convertible 15m-eu0xxx equipped with an AMD Ryzen 5 5500U processor (2.10 GHz, 6 cores) with Radeon Graphics, 8 GB of RAM, and Windows 11 Home Version 23H2 (OS build 22631.4460). These specifications represent a mid-range computing environment, demonstrating the protocol’s suitability for resource-constrained devices.

#### 6.2.2. Software Evaluation Results

The software evaluation focused on two primary metrics: communication overhead and computation overhead, as shown in the detailed performance results summarized in Table 2.

**Figure 5:**
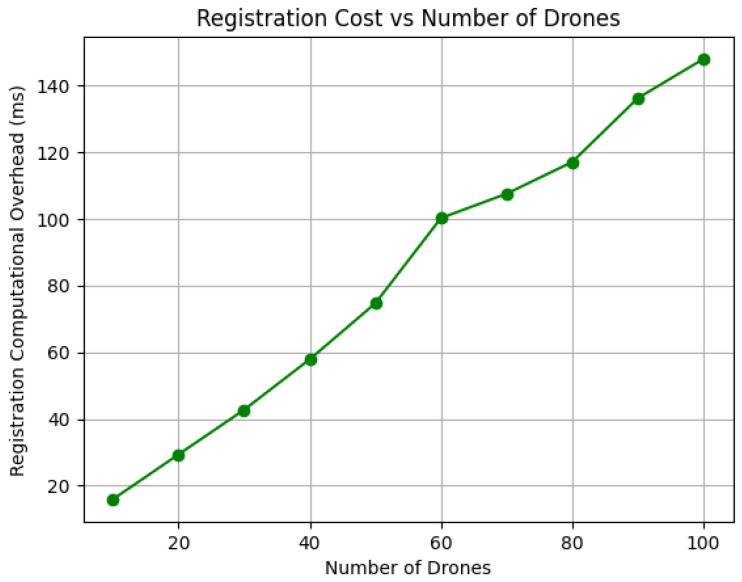
Registration computational overhead for different number of drones.

**Figure 6:**
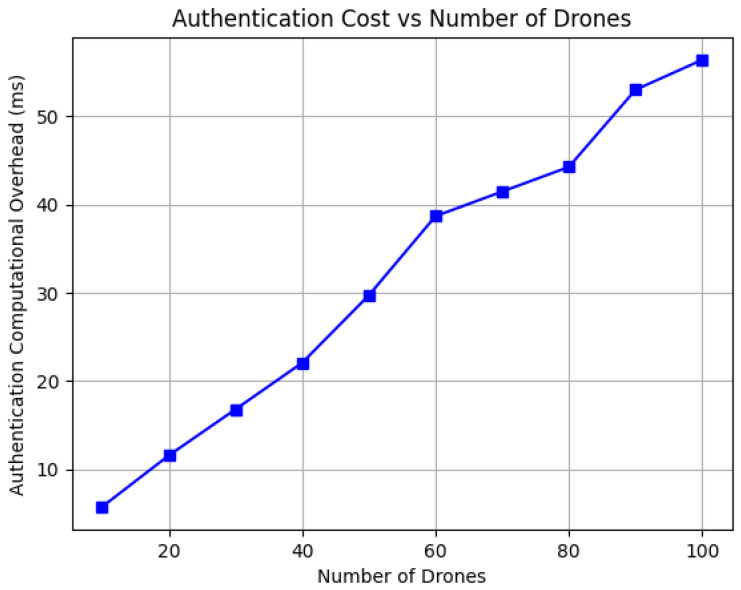
Authentication computational overhead for different number of drones.

- *Communication Overhead*: The communication overhead in our scheme is primarily influenced by the encryption technique employed. For this analysis, we used AES-256 encryption, which offers strong security and efficient implementation. The communication overhead reported in Table 2 quantifies only the additional overhead introduced by the proposed authentication protocol itself, namely, the extra control messages and cryptographic payloads exchanged during registration and authentication. Because the proposed scheme operates at the application/security layer, the reported values do not include lower-layer wireless stack overhead such as PHY/MAC framing, transport/network headers, channel contention, retransmissions, or propagation delay, which depend on the selected deployment technology rather than on the proposed scheme. Therefore, the reported 396 bytes for registration and 312.5 bytes for authentication should be interpreted as the incremental protocol-specific cost added to an existing drone communication link. In Python, encrypted messages are stored in a format that requires additional memory per digit, thereby increasing the overall communication cost. We calculated the average total communication overhead for all packets exchanged between the drone and the GSS during registration and authentication, after running the implementation 10 times. The registration phase required 396 bytes, which is higher because this phase distributes initial credentials and security parameters only once. In contrast, the authentication phase required 312.5 bytes since it performs periodic verification and therefore remains lightweight. Consequently, the complete protocol session requires 708.5 bytes, which remains small because the protocol transmits only hash outputs and symmetric encryption results instead of certificates or public-key parameters that are typically large in size.
- *Computation Overhead*: The computational overhead is assessed by measuring the time required for the scheme’s cryptographic operations, including registration and authentication. The performance of our scheme is evaluated using the SHA-256 hash function for key derivation and updates, and AES for encryption and decryption, thereby ensuring lightweight operations suitable for resource-constrained drones. The scalability of the scheme is illustrated in Figure 5 and Figure 6. As shown in Figure 5, the registration computation overhead increases gradually with the number of drones because each drone performs initialization operations once. Similarly, Figure 6 shows that the authentication overhead follows a comparable near-linear growth trend while remaining significantly lower, confirming that the protocol can support frequent real-time authentication. This efficiency is mainly due to the use of symmetric cryptography and hash functions instead of computationally expensive public-key operations. These results demonstrate that the protocol does not introduce heavy processing complexity and can efficiently support multi-drone swarm deployments.

The software evaluation demonstrates that the proposed scheme achieves low computational and communication overhead while preserving strong security properties.

### 6.3. Hardware Implementation

While the software evaluation demonstrates feasibility, the real strength of the proposed scheme lies in its hardware-assisted implementation. To further validate the practical deployability of the proposed scheme, a full hardware implementation was developed and evaluated on an FPGA platform.

#### 6.3.1. Experimental Setup

The proposed authentication scheme was implemented and synthesized on the Nexys A7-100T FPGA platform using Xilinx Vivado 2023.2. The design integrates three primary hardware modules: an XOR-based PUF for device identification, an AES-256 core for secure encryption and decryption, and a SHA-256 hashing unit for the cryptographic key generation process. These modules were interconnected through a lightweight control interface to ensure efficient operation while maintaining modularity. The entire design was clocked at 100 MHz, ensuring real-time performance suitable for resource-constrained IoD environments.

#### 6.3.2. Hardware Evaluation Results

The proposed authentication scheme was fully implemented and validated on a Nexys A7-100T FPGA to demonstrate its hardware feasibility and efficiency. The design integrates the AES-based encryption/decryption modules, SHA-256 hashing, and control logic into a unified architecture operating at 100 MHz. Synthesis results confirm that the implementation achieves low area and power overhead, utilizing only a small fraction of the available LUTs, registers, BRAMs, and DSP resources, while maintaining stable and reliable operation. The efficient clocking strategy and lightweight construction enable secure challenge–response authentication with minimal latency, making the proposed scheme well suited for resource-constrained platforms such as IoD and other embedded cyber-physical systems.

Table 1 summarizes the FPGA resource utilization for the implemented architecture. The proposed design consumed 7920 Slice LUTs and 9350 Slice Registers, corresponding to 12.49% and 7.37% of the available resources, respectively. This moderate utilization highlights the area efficiency of the implementation, leaving substantial resources available for additional on-chip functionalities such as communication and sensor interfaces. The Block RAM usage was limited to 8.88%, primarily utilized by the AES S-boxes and intermediate data buffers. Similarly, only 4.16% of DSP slices were occupied, reflecting that the design relies more on logical operations than arithmetic computation at a frequency of 100 MHz. The design required only two BUFG/clock buffers (6.25% utilization), indicating a simple and well-controlled clocking scheme that minimizes clock distribution overhead, reduces power consumption, and enhances timing stability across the FPGA fabric.

The power analysis revealed a total dynamic power consumption of approximately 182.5 mW, demonstrating the low power of the proposed hardware implementation. This power efficiency is particularly beneficial for UAV-based IoD systems where energy conservation is critical. The reported power value corresponds to the dynamic power consumption of the implemented authentication modules, excluding static FPGA and board-level power overhead. Furthermore, the operating frequency achieved of 100 MHz ensures that both the registration and authentication phases can be completed within milliseconds, satisfying the timing requirements for real-time drone communication. Overall, the FPGA implementation results confirm that the proposed authentication scheme achieves an effective balance between security, resource efficiency, and power consumption, making it well-suited for deployment in practical IoD networks.

### 6.4. Performance Evaluation of the Proposed Hybrid XOR-RO PUF

The proposed Hybrid XOR-RO PUF was implemented and tested on the Nexys A7-100T FPGA board operating at 100 MHz. A total of 500 challenge–response pairs (CRPs) were evaluated under different environmental conditions, including temperature variations between 20 °C and 85 °CC and a supply voltage range of 0.95–1.05 V. The PUF responses were analyzed for key performance indicators such as uniqueness, reliability, uniformity, and bit-aliasing, which collectively determine the security and stability of the design. Table 3 presents the statistical performance of the proposed design averaged across all measured CRPs.

The results indicate that the proposed design achieves near-ideal behavior in all major statistical parameters. The measured uniqueness of 49.12% demonstrates that responses from different PUF instances are highly decorrelated, ensuring strong device individuality. The reliability of 97.85% confirms that the generated responses remain consistent across temperature and voltage fluctuations. Similarly, uniformity and bit-aliasing are close to the ideal 50%, validating the balanced generation of logic ‘0’ and ‘1’ bits without significant bias.

For comparative evaluation, Table 4 summarizes the performance of the proposed Hybrid XOR-RO PUF against conventional Arbiter PUF and RO PUF architectures implemented under identical experimental conditions. As shown in the table, the proposed design achieves the highest uniqueness of 49.12%, which is closer to the ideal value of 50% than both the Arbiter PUF (47.68%) and the RO PUF (48.25%), indicating stronger device-to-device distinguishability. In terms of reliability, the Hybrid XOR-RO PUF significantly outperforms the baseline designs, achieving 97.85% reliability compared to 93.42% and 95.11% for the Arbiter and RO PUFs, respectively, thereby demonstrating enhanced robustness against temperature and voltage variations. Although the proposed design introduces a marginal increase in power consumption relative to the Arbiter PUF, it remains more power-efficient than the RO PUF while delivering substantially improved stability and security, confirming that the hybrid architecture offers a favorable trade-off among reliability, uniqueness, and hardware overhead.

**Figure 7:**
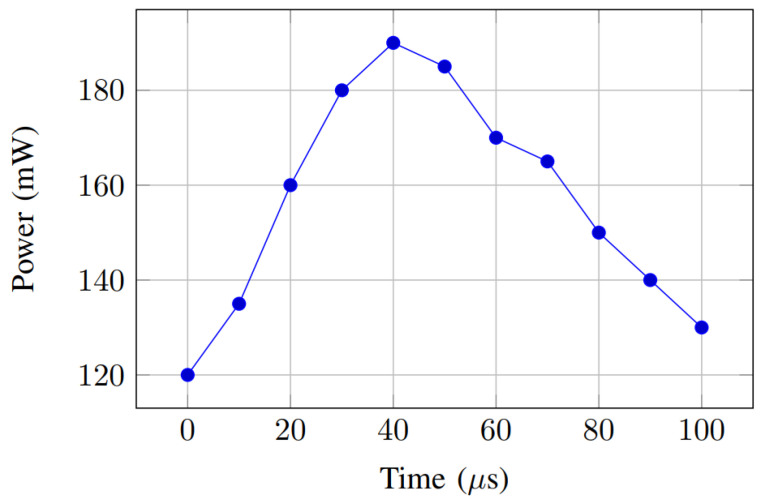
Power consumption profile of the proposed authentication scheme.

To evaluate the suitability of the proposed authentication scheme for energy-constrained UAV platforms, we analyzed its power and energy consumption during runtime. The results indicate an average dynamic power of 182.5 mW and an energy cost of approximately 86.4 μJ per authentication session. The temporal power profile, shown in Figure 7, reveals that peak consumption occurs during AES and SHA-256 operations, while the PUF module contributes minimal overhead. These findings confirm that the proposed design is energy-efficient and suitable for battery-powered drone applications.

For the inherent noise in PUF responses, the proposed design adopts a hardware-assisted reliability enhancement strategy without relying on helper data algorithms. The Hybrid XOR-RO architecture inherently improves stability by combining delay-based and frequency-based entropy sources, where the RO stage reduces sensitivity to environmental variations. Additionally, XOR-based response aggregation suppresses unstable bits through statistical averaging. During enrollment, only stable CRPs are selected through repeated measurements, and a tolerance-based matching scheme is applied during authentication to accommodate minor variations. This approach achieves high reliability while avoiding the overhead and security risks associated with helper data.

These results confirm that the hybrid combination of delay-based and frequency-based entropy sources significantly enhances the overall robustness of the PUF design. By leveraging XOR mixing of multiple arbiter and ring-oscillator outputs, the proposed PUF achieves a superior balance among randomness, stability, and hardware efficiency, making it a strong candidate for secure authentication in IoD and other lightweight IoT applications.

While the proposed Hybrid XOR-RO PUF builds upon established Arbiter and Ring Oscillator (RO) PUF principles, its novelty lies in the tight architectural integration and entropy coupling between delay-based and frequency-based sources within a unified FPGA-friendly framework. Unlike conventional XOR Arbiter PUFs that simply combine multiple arbiter outputs, the proposed design introduces cross-domain interaction, in which arbiter responses dynamically control RO pair activation and frequency comparison, effectively embedding temporal entropy into the spatial delay domain. This hybridization increases the dimensionality and nonlinearity of the challenge–response mapping, which is not present in standalone Arbiter or RO PUFs. The proposed design achieves higher reliability and greater uniqueness than recent baseline designs, while maintaining comparable power consumption, thereby providing a better trade-off between stability and hardware efficiency. Regarding modeling resistance, the XOR operation plays a critical role in enhancing security by introducing nonlinearity into the overall PUF response function. In a standard Arbiter PUF, the challenge–response behavior can often be approximated by linear additive delay models, making it vulnerable to machine-learning attacks such as logistic regression or support vector machines. However, when multiple independent response bits are XOR-combined, the resulting function becomes a higher-order nonlinear Boolean function, significantly increasing the complexity of the hypothesis space that an attacker must learn. In the proposed Hybrid XOR-RO PUF, this effect is further amplified because each XOR input is not purely delay-based but also influenced by RO frequency variations, which are inherently noisy and environment-dependent. Consequently, the combined response exhibits increased entropy and reduced correlation with the input challenge, thereby improving resistance against state-of-the-art modeling attacks. These properties collectively distinguish the proposed design from incremental extensions and position it as a robust and practical solution for secure hardware authentication.

## 7. Formal Security Verification Using ProVerif

To strengthen the security evaluation of the proposed authentication scheme, we conducted a formal verification using the *ProVerif* tool. *ProVerif* is a widely used automatic cryptographic protocol verifier that analyzes security properties under the Dolev–Yao adversary model, where the attacker can intercept, modify, and inject messages but cannot break secure cryptographic primitives [49]. In our model, the proposed protocol was formally represented by capturing the registration and authentication phases. The pseudo-identity generation, PUF-based challenge–response mechanism, and key derivation processes were explicitly modeled. Cryptographic operations were abstracted as follows: AES-256 encryption was modeled as a perfect symmetric encryption function, SHA-256 as a one-way hash function, and the PUF as a private function to represent its hardware-bound unclonability. The following security properties were verified: *Secrecy*: Ensuring that sensitive parameters such as the long-term key and drone identity are not accessible to an attacker. *Authentication*: Verifying that the ground station server correctly authenticates the drone.

**Figure 8:**
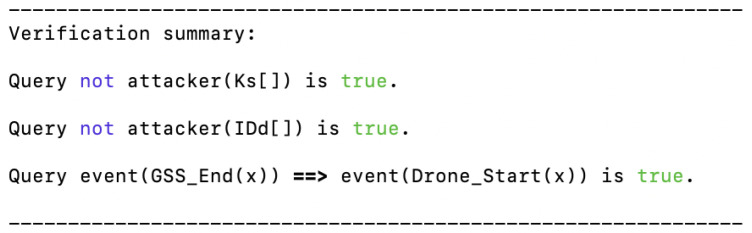
ProVerif verification results of the proposed authentication scheme.

The ProVerif analysis confirms that the attacker cannot derive the long-term shared key or the drone’s identity. Furthermore, the results shown in Figure 8 demonstrate that every successful authentication event at the server corresponds to a legitimate execution initiated by a valid drone. The verification results also indicate that the protocol provides one-way authentication, where the drone is authenticated by the ground station server. This is consistent with the design objectives of the proposed scheme and sufficient for many IoD scenarios in which the primary goal is to prevent unauthorized drones from accessing the network.

## 8. Conclusions and Future Work

This paper addressed the growing need for secure and efficient authentication mechanisms in IoD environments, where drones operate under strict computational, energy, and communication constraints. To tackle these challenges, a lightweight and privacy-preserving authentication scheme based on PUFs and efficient symmetric cryptographic primitives was proposed. By binding cryptographic keys to hardware-intrinsic characteristics through a PUF-assisted design, the scheme provides strong resistance to impersonation, cloning, and replay attacks while maintaining low computational and communication overhead. In addition, dynamically generated pseudo-identifiers enhance privacy by preventing drone tracking and concealing real identities during communication. To demonstrate the practicality of the proposed framework, both software-based and hardware-based implementations were developed and evaluated. The experimental results show that the scheme achieves a low communication overhead of 708.5 bytes and an average computation time of 18.87 ms, confirming its suitability for resource-constrained drone platforms. Furthermore, the hardware implementation on the Nexys A7-100T FPGA demonstrates efficient deployment with moderate resource utilization, low dynamic power consumption of approximately 182.5 mW, and stable real-time operation at 100 MHz. Although the present evaluation isolates the protocol-specific overhead introduced by the proposed scheme, future work will integrate this analysis with representative wireless-stack models for Wi-Fi, 4G/5G, and FANET deployments to quantify end-to-end latency under different drone communication environments. The proposed Hybrid XOR–RO PUF architecture combines delay-based and frequency-based entropy sources, achieving near-ideal statistical properties in terms of uniqueness, reliability, uniformity, and bit-aliasing, thereby validating the robustness of the hardware design under environmental variations. Despite these promising results, several limitations remain. The proposed scheme assumes secure initial provisioning of the long-term shared key during device deployment, which requires a trusted initialization process. This provision is intentionally scoped to pre-deployment or maintenance scenarios with stable connectivity, ensuring that the overhead of the registration phase does not affect the protocol’s real-time performance during active drone operations. In addition, the hardware implementation and evaluation were performed on a specific FPGA platform; therefore, further validation across diverse UAV hardware platforms and broader environmental conditions is necessary. Future work will focus on investigating the long-term reliability of the PUF under device aging and environmental variations, as well as exploring additional hardware optimizations to further reduce power consumption and resource utilization for low-power IoD platforms.

## Figures and Tables

**Table 1:** FPGA Resource Utilization of the Proposed Authentication Design on Nexys A7-100T.

Resource Type	Available	Used	Utilization (%)
Slice LUTs	63,400	7920	12.49%
Slice Registers	126,800	9350	7.37%
Block RAM (36Kb)	135	12	8.88%
DSP Slices	240	10	4.16%
BUFG/Clock Buffers	32	2	6.25%
Power (mW)	–	182.5	–
Operating Frequency	100 MHz		

**Table 2:** Performance analysis for Software implementation.

Metric	Registration	Authentication	Total
Number of messages	4	3	7
Number of keys	2	1	3
Communication Overhead (bytes)	396	312.5	708.5

**Table 3:** Performance Metrics of the Proposed Hybrid XOR–RO PUF.

Metric	Ideal Value	Proposed PUF Result
Uniqueness (%)	50	49.12
Reliability (%)	100	97.85
Uniformity (%)	50	48.76
Bit-Aliasing (%)	50	49.45
Challenge–Response Delay (ns)	–	23.4
Power Consumption (mW)	–	15.8

**Table 4:** Comparison of the Proposed Hybrid XOR–RO PUF with Existing Designs.

PUF Type	Uniqueness (%)	Reliability (%)	Power (mW)
Arbiter PUF	47.68	93.42	14.9
RO PUF	48.25	95.11	16.2
**Hybrid XOR–RO PUF**	**49.12**	**97.85**	**15.8**

## Data Availability

The data presented in this study are available on request from the corresponding author.
